# Reduced Access to Second Kidney Transplantation Among Adolescent Girls

**DOI:** 10.1016/j.ekir.2025.09.015

**Published:** 2025-09-16

**Authors:** Alexandra C. Bicki, Sang M. Nguyen, Gabriela Accetta Rojas, David V. Glidden, Barbara Grimes, Elaine Ku

**Affiliations:** 1 Division of Pediatric Nephrology, Department of Pediatrics, University of California, San Francisco; San Francisco, California, USA; 2 Department of Pediatrics, University of California, San Francisco; San Francisco, California, USA; 3 Department of Pediatrics, Nemours Children’s Health, Wilmington, Delaware, USA; 4 Department of Epidemiology and Biostatistics, University of California, San Francisco, San Francisco, California, USA; 5 Division of Nephrology, Department of Medicine, University of California, San Francisco, San Francisco, California, USA

**Keywords:** adolescents, graft failure, transplantation

## Introduction

There is a well-documented evidence of a disparity in access to first kidney transplantation by sex, with adult women being less likely to receive a transplant than men. This disparity is persistent even when considering the known sensitizing event of pregnancy.^1^ Adolescent girls who have never been pregnant also have lower access to first kidney transplantation, particularly from a living donor.^2^ However, graft survival may depend on a number of factors, such as sex of the donor kidney^3^ in addition to issues surrounding medication adherence that could affect readiness for a subsequent transplant.^4^ Here, we aimed to determine whether women and girls continue to have lower access to transplantation than men and boys after first graft failure, with the inclusion of adolescents who are highly likely to need a second transplant during their lifetime.

## Results

The cohort and analytic approach is described in the Supplementary Methods. We included 72,319 individuals in the study. The median age at graft failure was 54 years (interquartile range [IQR]: 42–64 years); 60% were men and 48% were of non-Hispanic White race (Table 1). Median vintage of the first graft was 7.0 years (IQR: 3.0–12.0 years). Over a median follow-up time of 1.9 years (IQR: 0.5–4.4 years) after graft failure, 28,460 individuals died (39%) and 21,204 individuals (29%) received a second transplant.

**Table 1:** Characteristics of individuals ≥ 13 years of age with first kidney allograft failure (USRDS 2006–2019)

*N* (%) or median (IQR)	Overall (*N* = 72,319)	Men (*n* = 43,460, 60.1%)	Women (*n* = 28,859, 39.9%)
Age at first graft failure (yr)	54.0 (41.9–63.8)	54.6 (43.0–64.1)	53.0 (40.4–63.3)
13 to < 18	1031 (1.4%)	599 (1.4%)	432 (1.5%)
18 to < 40	14,820 (20.5%)	8214 (18.9%)	6606 (22.9%)
40 to < 65	40,490 (56.0%)	24,751 (57.0%)	15,739 (54.5%)
≥ 65	15,978 (22.1%)	9896 (22.8%)	6082 (21.1%)
BMI at first graft failure (kg/m^2^)	26.2 (22.5-30.9)	26.3 (22.9–30.7)	26.0 (21.9–31.3)
cPRA at first graft failure (%)	74.32 (0.0–98.37)	69.68 (0.0–97.9)	80.43 (0.0–98.8)
Kidney failure etiology			
Glomerulonephritis	20,873 (28.9%)	11,483 (26.4%)	9390 (32.5%)
Diabetes	18,003 (24.9%)	11,241 (25.9%)	6762 (23.4%)
Hypertension	14,261 (19.7%)	9492 (21.8%)	4769 (16.5%)
Other/missing/unknown	11,655 (16.1%)	6937 (16.0%)	4718 (16.3%)
Cystic kidney diseases	4810 (6.7%)	2631 (6.1%)	2179 (7.6%)
Other urologic etiologies	2717 (3.8%)	1676 (3.9%)	1041 (3.6%)
Race/Ethnicity			
Asian	2963 (4.1%)	1666 (3.8%)	1297 (4.5%)
Hispanic	10,185 (14.1%)	6103 (14.0%)	4082 (14.1%)
Non-Hispanic Black	22,834 (31.6%)	13,591 (31.3%)	9243 (32.0%)
Non-Hispanic White	35,023 (48.4%)	21,384 (49.2%)	13,639 (47.3%)
Other/missing/unknown	1314 (1.8%)	716 (1.6%)	598 (2.1%)
Blood group			
O	33,132 (45.8%)	19,610 (45.1%)	13,522 (46.9%)
A	25,654 (35.5%)	15,577 (35.8%)	10,077 (34.9%)
B	9496 (13.1%)	5831 (13.4%)	3665 (12.7%)
AB	3283 (4.5%)	1991 (4.6%)	1292 (4.5%)
Missing/unknown	754 (1.0%)	451 (1.0%)	303 (1.0%)
Insurance status			
Medicare/Medicaid	29,201 (40.4%)	17,015 (39.2%)	12,186 (42.2%)
Private	26,753 (37.0%)	16,455 (37.9%)	10,298 (35.7%)
None/missing/unknown	16,365 (22.6%)	9990 (23.0%)	6375 (22.1%)
Calendar period of first graft failure			
2006–2014	44,817 (62.0%)	26,809 (61.7%)	18,008 (62.4%)
2015–2019	27,502 (38.0%)	16,651 (38.3%)	10,851 (37.6%)

BMI, body mass index; cPRA, calculated panel reactive antibody; IQR, interquartile range; USRDS, United States Renal Data System.

BMI was available for 85.0% of the cohort (61,456 patients: 36,788 men, and 24,668 women); 90% of patients had BMI available within 3.7 months of graft failure. cPRA at the time of graft failure was available for 50.0% of the cohort (36,215 patients: 21,511 men and 14,704 women). Here, IQR indicates 25 percentile to 75 percentile.

Of those with a known donor type, 14,343 individuals received a second transplant from a deceased donor (68% of all second transplants) and 6846 individuals received a second transplant from a living donor (32% of all second transplants). Among those who received preemptive transplant (*n* = 3523, 4.9% of the cohort), there was no difference in receipt of a second transplant by sex (*P* = 0.29). However, among adolescents (aged 13 to < 18 years at first graft failure), boys were more likely to receive a preemptive second transplant (5.0% of all adolescents) than girls (2.2%, *P* = 0.041).

**Figure 1:**
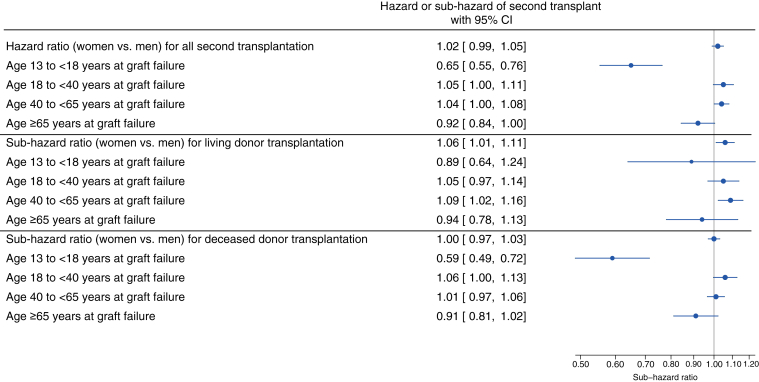
Hazard or subhazard of second kidney transplant in women versus men, stratified by age at first graft failure (USRDS, 2006–2019). Models were adjusted for age and body mass index at first graft failure, race/ethnicity (Asian, Hispanic, Non-Hispanic Black, Non-Hispanic White, Other), neighborhood income, calendar period (before vs. after 2014 Kidney Allocation System changes), insurance type, ABO blood group, and comorbidities. CI, confidence interval; USRDS, United States Renal Data System.

The overall hazard of second transplant was not different by sex (hazard ratio: 1.02, 95% confidence interval: 0.99–1.05; Figure 1) and did not differ by type of donor (living vs. deceased). There was an interaction between age and sex (*P*_int_ < 0.001) such that adolescent girls had reduced access to a second transplant than adolescent boys (hazard ratio: 0.65, 95% CI 0.55–0.76; Figure 1). This finding was particularly prominent for the outcome of receipt of a second deceased donor transplant in adolescent girls compared with boys (sub-hazard ratio: 0.59, 95% confidence interval: 0.49–0.72). The difference in rates of second deceased donor transplant by sex was not observed among older age groups.

Among those with calculated panel reactive antibody available at the time of second waitlisting, adolescent girls aged 13 to < 18 years at first graft failure had higher calculated panel reactive antibody at time of first graft failure than same-aged boys (84% vs. 75%, respectively, *P* = 0.006; Supplementary Table S1). However, there was no significant overall interaction between sex and calculated panel reactive antibody on the outcome (*P*_int_ = 0.65).

We performed a number of exploratory analyses among the subgroup of those with graft failure in adolescence. There were no interaction effects between sex and race or ethnicity (*P*_int_ = 0.92), sex and Kidney Allocation System period (*P*_int_ = 0.86), or sex and the first-kidney donor type (living vs. deceased, *P*_int_ = 0.42). There were considerable differences in the vintage of the first graft, such that girls with graft failure in adolescence had a graft vintage of only 3.2 years (IQR: 1.4–6.7), which was 56% shorter than that of same-aged boys (7.3 years, IQR: 2.7–11.9; Supplementary Figure S1, *P* < 0.001). There was no difference by sex in whether adolescents with graft failure were subsequently relisted (*P* = 0.14).

In sensitivity analysis, we performed Fine-Gray analyses treating death as a competing risk for the primary analysis of any second transplantation; results were similar to the overall findings (Supplementary Table S2).

## Discussion

Adolescent girls who develop graft failure had a nearly 35% lower rate of receiving a second transplant than boys. This disparity appears to be driven by reduced access to deceased donor transplantation. This association was not observed in other age groups and not explained by differences in sensitization. Although pregnancy-related sensitization may contribute to disparities in access to transplantation by sex among adults, this seems less likely to be a plausible explanation among adolescent girls. Other studies have suggested that reduced awareness of the waitlisting, evaluation, and transplant process may contribute to sex disparities in access to kidney transplantation^5^; however, given these families’ experience in navigating the process during the patient’s first transplant, this seems less likely to be an explanation for these findings.

Alternative explanations for the observed disparity could relate to differences by sex with regard to medication adherence and mental health comorbidities, which may both influence an individual’s potential for graft rejection and successful relisting for—and subsequent receipt of—a second transplant. We did identify significant differences in the duration of survival of the first graft in the 13 to < 18-years age group, with girls having a median first-graft vintage of only 3 years. This difference extended into young adulthood, with women having reduced first-graft survival compared with same-aged men. We speculate that the short initial graft survival among adolescent girls is likely due to medication nonadherence and rejection given the high-quality organs received by pediatric patients, and could be a potential explanation for the longer time to a second transplant in this population. However, data from a multicenter study of pediatric and young adult kidney transplant recipients conflict as to whether medication adherence consistently varies by sex.^4^ The prevalence of mental health diagnoses or availability of social support may also differ by sex among adolescents with chronic kidney disease. For example, data from the Chronic Kidney Disease in Children cohort study have demonstrated that girls with chronic kidney disease have a higher prevalence of formally diagnosed depression than boys^6^; however, it is unclear if this disparity persists to the same extent after transplantation, since—among adults—mental health generally improves after transplantation.^7^

Major limitations of this study include missing data on the specific cause of graft failure (e.g., missing in 41% of the adolescents), missing data on calculated panel reactive antibody at the time of graft failure, and the potential presence of residual confounding. We do not have access to detailed estimated glomerular filtration rate trajectories prior to graft failure and are unable to determine if the rate of progression to graft failure differed by sex and could have contributed to the differential access of adolescent girls versus boys to a second preemptive transplant. Patients who received a preemptive second transplant were included in the overall cohort but contributed minimal follow-up time. However, because more adolescent boys received preemptive second transplant than girls, their inclusion likely resulted in a conservative estimate of the observed sex disparity.

The finding related to modestly higher likelihood of living donor second transplant among women aged 40 to < 65 years, as compared with same-aged men is interesting, though our data are limited in our ability to identify the exact mediators of this finding. Previous literature has suggested that women are less likely to be referred for transplant and to consider themselves eligible candidates for transplantation. We speculate that younger women who have already received a first transplant and overcome known barriers to a first transplant (e.g., referral and self-perceptions of their eligibility for transplantation) are a selected group of individuals who are more likely to self-advocate and remain proactive in attaining a second transplant. This finding did not extend to older women, though previous studies have suggested that provider perceptions of frailty in women may serve as a barrier.^8^

## Conclusion

Adolescent girls with graft failure had reduced access to a second kidney transplant compared with adolescent boys. Failing to reach transplantation, particularly during the critical developmental period of adolescence and when adolescents are prone to graft failure, can have long-term medical and psychosocial consequences.^9^ Further studies are needed to understand the reasons for this observation so that these sex disparities in retransplantation can be addressed.

## Disclosure

EK is an Associate Editor at the American Journal of Kidney Diseases and has received funding from Natera in the form of grants. All the other authors declared no competing interests.

## Supplementary Material

Supplementary File (PDF) **Supplementary Methods. Supplementary References. Figure S1.** Vintage of the first allograft among individuals with kidney allograft failure by age at first graft failure (median and IQR, yrs). **Table S1.** Characteristics of individuals aged 13 to < 18 years with kidney allograft failure, USRDS 2006–2019. **Table S2.** Sensitivity analysis comparing Cox model hazard ratios to Fine Gray subhazard ratios with death as competing event.

## Footnotes

Supplementary File (PDF)

**Supplementary Methods.**

**Supplementary References.**
**Figure S1.** Vintage of the first allograft among individuals with kidney allograft failure by age at first graft failure (median and IQR, yrs). **Table S1.** Characteristics of individuals aged 13 to < 18 years with kidney allograft failure, USRDS 2006–2019. **Table S2.** Sensitivity analysis comparing Cox model hazard ratios to Fine Gray subhazard ratios with death as competing event.
