# Supplementary material for: Reduced Access to Second Kidney Transplantation Among Adolescent Girls
Source: Kidney Int Rep. 2025 Sep 16;10(12):4268–71. doi: 10.1016/j.ekir.2025.09.015 (PMC12712456; doi:10.1016/j.ekir.2025.09.015)
Supplement: Supplementary File (PDF) — Supplementary Methods. Supplementary References. Figure S1. Vintage of the first allograft among individuals with kidney allograft failure by age at first graft failure (median and IQR, yrs). Table S1. Characteristics of individuals aged 13 to < 18 years with kidney allograft failure, USRDS 2006–2019. Table S2. Sensitivity analysis comparing Cox model hazard ratios to Fine Gray subhazard ratios with death as competing event. [file mmc1.pdf]

Supplementary Methods

Study Population

We performed a retrospective cohort study of patients ≥13 years of age who experienced first kidney allograft failure from January 1, 2006, to December 31, 2019 based on data from the United States Renal Data System (USRDS).[S1] We included those who received a preemptive transplant in the cohort. The primary predictor was sex (as reported on the Centers for Medicare and Medicaid Services (CMS) Form-2728, End Stage Renal Disease Medical Evidence Report, which is completed within 45 days of kidney replacement therapy initiation and affirmed by dialysis provider and the patient/guardian). The primary outcome was receipt of a second kidney transplant (overall, and also by living donor vs. deceased donor source of the second transplant). We employed age at graft failure rather than age at second waitlisting to ensure inclusion of all patients with graft failure and avoid restricting the analysis to only those who were relisted (who may be a select group of patients).

Patient demographic characteristics (age at first graft failure, race/ethnicity, and ABO blood group), kidney failure etiology, insurance status at kidney failure onset (Medicare/Medicaid, private, or none), median neighborhood income by ZIP code at kidney failure onset, body mass index (BMI) within one year of graft failure, medical comorbidities (chronic obstructive pulmonary disease, diabetes mellitus, peripheral vascular disease, heart failure, hypertension, non-ambulatory status, stroke) and calculated panel reactive antibody (cPRA) at the time of graft failure were abstracted from the Medical Evidence Report and USRDS Patients file. The patient's ZIP code was ascertained using the Patients file to determine median neighborhood income according to the American Community Survey. Race and ethnicity were categorized as Asian, Hispanic, non-Hispanic Black, non-Hispanic White, or

Other/Unknown (including Alaska Native, American Indian, Arabian, Asian, Middle Eastern, Native Hawaiian, Pacific Islander, and unknown) and were based on data in the Patients file.

Statistical Analysis

Graft failure was defined as the need to restart dialysis or receipt of a preemptive second kidney transplant. Baseline characteristics of the study population were reported as number (percentage) or median (with interquartile range, IQR), as appropriate.

We used adjusted Cox proportional hazard models to examine the association between the primary predictor of sex and the primary outcome of receipt of a second transplant. All analyses were adjusted for age and BMI at graft failure, sex, race/ethnicity, median neighborhood income, calendar period (before vs. after 2014 Kidney Allocation System (KAS) changes), insurance type, ABO blood group, and comorbidities. Covariates were selected based on their associations with transplant access and outcomes and in consideration of models used in prior literature.[S2, S3] Fine-Gray models accounting for the competing risk of death in evaluating the outcome of any second transplantation were employed in sensitivity analysis.

Multiple iterative chained imputation equations were used to impute BMI and median income, with 25 imputations. BMI was missing in 14.8% of patients (n=10732), median income was missing in 1.0% (n=738) of patients, and both BMI and median income were missing in 0.2% of patients (n=131). Age at graft failure, sex, race/ethnicity, blood group, kidney failure etiology, insurance status, and comorbidities were used as predictors in the imputation regression models.

For the outcome of deceased donor transplantation, Fine-Gray models accounting for the competing risks of living donor transplant or death were used. For the outcome of living donor

transplantation, Fine-Gray models accounting for the competing risks of deceased donor transplant or death were used.

We tested for interaction between age categories or calculated panel reactive antibody (cPRA) at the time of first graft failure with sex. Among adolescents, we also tested for interaction between first donor type, race/ethnicity, KAS period and sex. Interactions were considered statistically significant and subgroup analyses were pursued if the p-value for the interaction term was <0.05. We performed exploratory analyses to evaluate differences in graft vintage by age at graft failure and sex subgroup. Analyses were conducted in SAS version 9.4. The Institutional Review Board at the University of California, San Francisco deemed this study exempt human subjects research.

Supplemental Table 1. Characteristics of individuals 13 to <18 years of age with kidney allograft failure, USRDS 2006-2019

**N (%) unless otherwise stated**	Overall (N = 1031)	Boys (n = 599, 58.1%)	Girls (n = 432, 41.9%)
Age at first allograft failure (median and interquartile range, years)	16 (15-17)	16 (15-17)	16 (15-17)
BMI at first allograft failure (mean ± standard deviation, kg/m^2)	23.1 ± 8.4	22.4 ± 5.7	24.1 ± 11.1
cPRA at first allograft failure (median and interquartile range,)	81% (3-99%)	75% (0-97%)	84% (24-99%)
Kidney failure etiology			
Other/Unknown/Missing	424 (41.2%)	259 (43.2%)	165 (38.2%)
Glomerulonephritis	318 (30.8%)	143 (23.9%)	175 (40.5%)
Urologic etiologies	215 (20.9%)	153 (25.5%)	62 (14.4%)
Cystic kidney diseases	74 (7.2%)	44 (7.3%)	30 (6.9%)
Race/Ethnicity			
Hispanic	222 (21.5%)	108 (18.0%)	114 (26.4%)
Non-Hispanic Black	297 (28.8%)	180 (30.1%)	117 (27.1%)
Non-Hispanic White	476 (46.2%)	293 (48.9%)	183 (42.4%)
Asian/Other/Unknown/Missing	36 (3.4%)	18 (3.0%)	18 (4.1%)
Blood Group			
O	507 (49.2%)	303 (50.6%)	204 (47.2%)
A	343 (33.3%)	188 (31.4%)	155 (35.9%)
B	133 (12.9%)	82 (13.7%)	51 (11.8%)
AB or Unknown/Missing	48 (4.7%)	26 (4.3%)	22 (5.1%)
Insurance type			
Medicare/Medicaid	571 (55.4%)	311 (51.9%)	260 (60.1%)
Private	263 (25.5%)	163 (27.2%)	100 (23.1%)
None/Unknown/Missing	197 (19.1%)	125 (20.9%)	72 (16.7%)
Calendar period of first graft failure			
2006-2014	721 (69.9%)	396 (66.1%)	325 (75.2%)
2015-2019	310 (30.1%)	203 (33.9%)	107 (24.8%)

Note: Over the observation period, 1031 individuals experienced first graft failure at age 13 to <18. BMI was available for 96.7% of these adolescents (997 patients, 581 boys and 416 girls). cPRA at first graft failure was available for 81% of these adolescents (831 patients, 493 boys and 338 girls). Some kidney failure etiologies, race/ethnicity, and blood group categories are collapsed to comply with USRDS reporting requirements, such that all cells have ≥11 individuals.

Abbreviations: BMI, body mass index; cPRA, calculated panel reactive antibody

Supplemental Table 2. Sensitivity analysis comparing Cox model hazard ratios to Fine-Gray sub-hazard ratios with death as competing event

Age at First Graft Failure Subgroup	Cox model Hazard Ratio (95% CI), p-value	Fine-Gray Sub-hazard Ratio (95% CI), p-value
13-<18 years	0.65 (0.55-0.76), p<0.001*	0.66 (0.56-0.78), p<0.001*
18-<40 years	1.05 (1.00-1.11), p=0.033*	1.05 (1.00-1.10), p=0.072
40-<65 years	1.04 (1.00-1.08), p=0.047*	1.04 (1.00-1.08), p=0.033*
≥65 years	0.92 (0.84-1.00), p=0.077	0.93 (0.85-1.03), p=0.157

*p<0.05

Supplemental Figure 1. Vintage of the first allograft among individuals with kidney allograft failure by age at first graft failure (median and IQR, years)

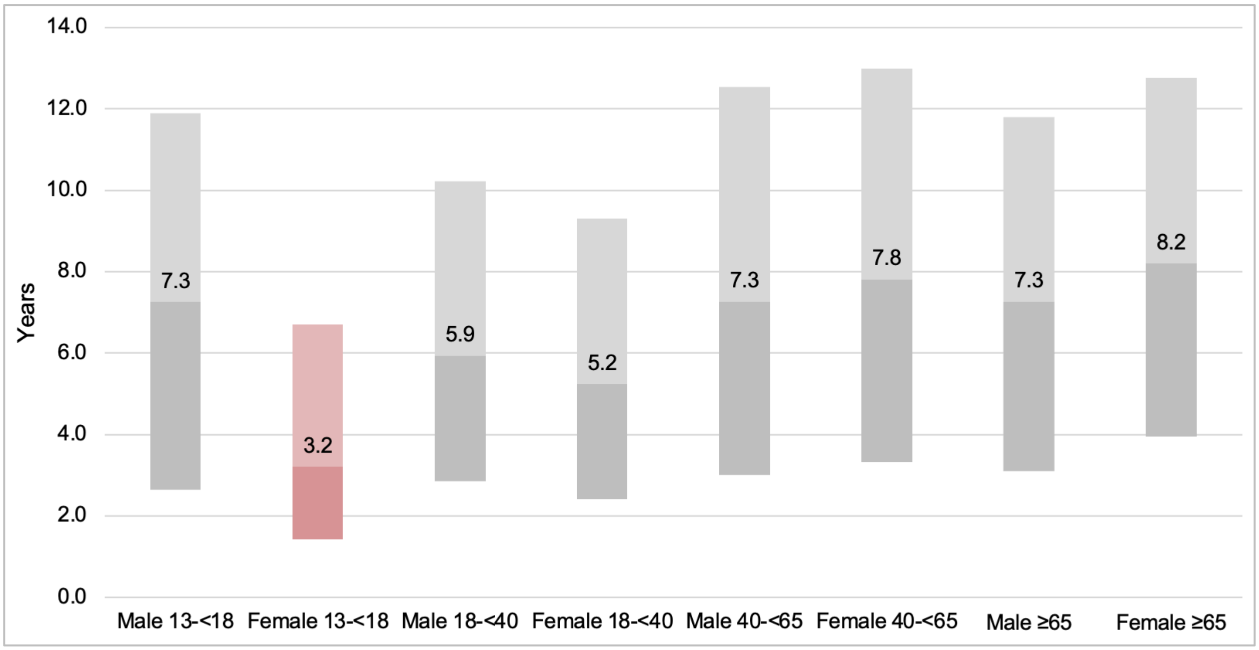

Supplemental References

S1. U.S. Renal Data System. 2024 USRDS Annual Data Report: Epidemiology of Kidney Disease in the United States. National Institutes of Health, National Institute of Diabetes and Digestive and Kidney Diseases, Bethesda, MD, 2024.

S2. Harding JL, Di M, Pastan SO, et al. Sex/Gender-Based Disparities in Early Transplant Access by Attributed Cause of Kidney Disease–Evidence from a Multiregional Cohort in the Southeast United States. Kidney Int Rep. 2023;8(12):2580-2591. doi:10.1016/j.ekir.2023.09.010

S3. Sy J, Streja E, Grimes B, Johansen KL. The Marginal Cost of Frailty Among Medicare Patients on Hemodialysis. Kidney Int Rep. 2019;5(3):289-295. doi:10.1016/j.ekir.2019.11.020
